# Multidimensional Genetic Analysis of Repeated Seizures in the Hybrid Mouse Diversity Panel Reveals a Novel Epileptogenesis Susceptibility Locus

**DOI:** 10.1534/g3.117.042234

**Published:** 2017-06-15

**Authors:** Russell J. Ferland, Jason Smith, Dominick Papandrea, Jessica Gracias, Leah Hains, Sridhar B. Kadiyala, Brittany O’Brien, Eun Yong Kang, Barbara S. Beyer, Bruce J. Herron

**Affiliations:** * Department of Neuroscience and Experimental Therapeutics, Albany Medical College, Albany, New York 12208; † Department of Neurology, Albany Medical College, Albany, New York 12208; ‡ Department of Biological Sciences, Center for Biotechnology and Interdisciplinary Studies, Rensselaer Polytechnic Institute, Troy, New York 12180; § Wadsworth Center, New York State Department of Health, Albany, New York 12201; ** Department of Biomedical Sciences, School of Public Health, University at Albany, State University of New York, New York 12201; †† Department of Computer Science, University of California, Los Angeles, California 90095

**Keywords:** genetics, epilepsy, preclinical model, neuronal plasticity, complex traits

## Abstract

Epilepsy has many causes and comorbidities affecting as many as 4% of people in their lifetime. Both idiopathic and symptomatic epilepsies are highly heritable, but genetic factors are difficult to characterize among humans due to complex disease etiologies. Rodent genetic studies have been critical to the discovery of seizure susceptibility loci, including *Kcnj10* mutations identified in both mouse and human cohorts. However, genetic analyses of epilepsy phenotypes in mice to date have been carried out as acute studies in seizure-naive animals or in Mendelian models of epilepsy, while humans with epilepsy have a history of recurrent seizures that also modify brain physiology. We have applied a repeated seizure model to a genetic reference population, following seizure susceptibility over a 36-d period. Initial differences in generalized seizure threshold among the Hybrid Mouse Diversity Panel (HMDP) were associated with a well-characterized seizure susceptibility locus found in mice: *Seizure susceptibility 1*. Remarkably, *Szs1* influence diminished as subsequent induced seizures had diminishing latencies in certain HMDP strains. Administration of eight seizures, followed by an incubation period and an induced retest seizure, revealed novel associations within the calmodulin-binding transcription activator 1, *Camta1*. Using systems genetics, we have identified four candidate genes that are differentially expressed between seizure-sensitive and -resistant strains close to our novel *Epileptogenesis susceptibility factor 1* (*Esf1*) locus that may act individually or as a coordinated response to the neuronal stress of seizures.

Epilepsies in humans are highly heritable syndromes that are modified by complex interactions between genes and the environment. In the US, ∼2.2 million people will develop some form of epilepsy during their lifetime (England *et al.* 2012). Despite diverse treatment options, largely focused on seizure suppression, individuals with epilepsy suffer multiple quality of life issues, refractory seizures, comorbid complications, and a higher incidence of sudden death (Gupta *et al.* 2016). Understanding interactions between genetic seizure susceptibility loci and environmental stressors induced by repeated seizures will uncover novel mechanisms of epilepsy pathogenesis. This information will improve therapeutic options by focusing treatment on epileptogenesis (Ferraro 2016).

Identification of gene by environment interactions for individuals with epilepsy is limited by complex disease etiologies and the inability to identify at risk populations prior to disease onset. Consequently, the majority of epilepsy genome-wide association studies (GWAS) carried out to date compare affected populations to seizure-naive controls rather than following epilepsy progression in a population from a seizure-naive to an epileptic state (International League Against Epilepsy Consortium on Complex Epilepsies 2014). Experimental seizure paradigms can overcome such limitations to discover the genetic basis of epileptogenesis.

Preclinical epilepsy models have implicated multiple gene mutations as contributors to seizure disorders. This success is due in part to strong genetic influences that segregate among rodent models of seizure susceptibility (Engstrom and Woodbury 1988; Kosobud and Crabbe 1990). Unfortunately, most of these models utilized either acute seizure measurements or status epilepticus as quantitative measures of seizure susceptibility (Grone and Baraban 2015). Alternatively, animal models that measure epileptogenesis and other models of brain plasticity are typically designed to minimize genetic background effects (Grone and Baraban 2015). Notably, while numerous hyper-excitability factors are now known, genetic factors influencing epileptogenesis remain largely uncharacterized.

We have utilized a repeated-flurothyl-induced seizure model delivered to the Hybrid Mouse Diversity Panel (HMDP) to identify genetic susceptibility factors that modify epileptogenesis (Papandrea *et al.* 2009). Genetic differences in initial flurothyl-induced seizures were consistent with previous studies showing the strongest quantitative trait loci (QTL) mapping to the *Szs1* locus (Ferraro *et al.* 1997, 2004; Chaix *et al.* 2007). Surprisingly, as additional seizures were administered to this population, the effects of *Szs1* diminished, and genetic associations on distal chromosome 4 garnered influence over seizure susceptibility. We attribute the shift in susceptibility to seizure-related changes in brain plasticity that are modified by gene(s) that may mitigate the effects of *Szs1*. This hypothesis is supported by interactions between *Szs1* and our novel epileptogenesis susceptibility locus on chromosome 4; *Epileptogenesis susceptibility factor 1* (*Esf1*). Systems genetics analysis of the *Esf1* region has uncovered a coregulated set of expression QTL (eQTL) that have been implicated in neurological stress responses that are now also implicated in epileptogenesis.

## Materials and Methods

### Animals

The rationale for using the HMDP and its advantages with respect to statistical power and genetic resolution have been previously described (Bennett *et al.* 2010). The inbred strains of mice that were used for this study include the following: Balb/cJ (Balb; *n* = 8); BTBR *T^+^Ltpr3^tf^*/J (BTBR; *n* = 6); BXD#/TyJ (30 strains, average *n*/strain ≤6); BXD##/RwwJ (18 strains, average *n*/strain ≤4); C57BL/6J (B6; *n* = 10); C57BL/10SNJ (10SNJ; *n* = 12); C57BL/10J (10J; *n* = 8); C57BL/6NJ (6NJ; *n* = 16); C57BLKS/J (KS/J; *n* = 15); CAST/EiJ (CAST; *n* = 6); CBA/J (CBA; *n* = 17); CE/J (CE; *n* = 11); DBA/2J (D2; *n* = 14); FVB/NJ (FVB; *n* = 12); LG/J (LG; *n* = 19); MRL/MpJ (MRL; *n* = 6); NOD/ShiLtJ (NOD; *n* = 13); NZW/LacJ (NZW; *n* = 10); PWD/PhJ (PWD; *n* = 9), and Sm/J (Sm; *n* = 13). Male and female mice were obtained from the Jackson Laboratories (Bar Harbor, ME) and (1) were acclimated to the animal facility for 1 wk before seizure testing commenced, or (2) were further bred at the Wadsworth Center and exposed to the repeated-flurothyl seizure model. Mice were housed on a 12 hr light–dark cycle with *ad libitum* access to food and water.

A [B6×D2] F2 cross was generated by intercrossing [B6×D2] F1 parents. Both male and female F2 mice were exposed to the repeated-flurothyl seizure model as described below (Papandrea *et al.* 2009; Kadiyala *et al.* 2016). Mice (*n* = 150) from this cohort were genotyped with the Mouse Universal Genotyping Array (http://genomics.neogen.com/en/mouse-universal-genotyping-array) and mapping (regression analysis) was performed using R/qtl (http://www.rqtl.org; Broman *et al.* 2003).

All testing was performed under approval of the Institutional Animal Care and Use Committees of the Wadsworth Center, Albany Medical College, and Rensselaer Polytechnic Institute in accordance with the National Institutes of Health’s Guide for the Care and Use of Laboratory Animals.

### Repeated-flurothyl seizure model

Six- to seven-week-old male and female mice were placed in a closed chamber and exposed to an inhaled 10% flurothyl solution [bis(2,2,2-trifluoroethyl) ether diluted in 95% ethanol] infused via a syringe pump onto a gauze pad suspended at the top of the chamber at a rate of 100 μl/min (Papandrea *et al.* 2009; Kadiyala *et al.* 2015; Ferland, 2017). Once a generalized seizure was observed, indicated by a loss of postural control, the top of the chamber was removed to stop flurothyl exposure and allowing the animal to recover. The latency to the loss of postural control (measured in seconds) was recorded as the generalized seizure threshold (GST). Mice received a single flurothyl-induced seizure once a day for 8 consecutive days during the induction phase. The induction phase was followed by a 28-d incubation phase where no induced seizures were administered. After the incubation phase, mice were given one additional flurothyl-induced retest seizure. The reduction in daily GST in certain HMDP strains over the induction phase (kindling) was quantified by plotting the eight induction phase GSTs over the seizure number and calculating the slope of these data.

### Efficient Mixed-Model Association (EMMA)

EMMA is a statistical test for association mapping that can correct for genetic relatedness due to population structure (Bennett *et al.* 2010). The calculations for genome-wide false discovery rate of 5% in the HMDP is *P* < 4.4 × 10^−6^ (Bennett *et al.* 2010). The implementation of EMMA used here is available online at http://mouse.cs.ucla.edu/emma. All association studies were carried out with male seizure data. A limited set of female data for select HMDP strains were collected, but did not show sufficient differences from their male counterparts to warrant further study in a genome-wide analysis. These data are available upon request.

### RNA extraction

Brains (*n* = 5 per group) were isolated from 11- to 12-wk-old B6 and D2 seizure-naive or seizure-exposed mice 90 min after completion of the repeated-flurothyl model as in Kadiyala *et al.* (2015). Brains were incubated in RNAlater at 4° overnight and subsequently stored at −20° until dissection. Cerebellum and hippocampus were dissected from whole brains, weighed, and processed with the RNeasy Lipid Tissue Mini Kit (Qiagen) using on-column DNAse digestions during mRNA purification. Total RNA concentrations were measured via Nanodrop and samples were aliquoted and stored at −80°.

### cDNA synthesis

One microgram of total RNA was primed with random hexamers using the NEB Protoscript first strand cDNA synthesis kit per the manufacturer’s protocol. Ten-fold dilutions were prepared for quantitative PCR (qPCR).

### Quantitative PCR

Relative RNA expression levels were quantified using the following Primetime premade 5′ nuclease assays from Integrated DNA Technologies: *Actb* (Loading control, NM_007393, exons 4–5, Mm.PT.58.33257376); *Per3* (NM_011067, exons 4–6, Mm.PT.58.12973804); Park7 (NM_020569, exons 2–3, Mm.PT.58.7064397); *Camta1* (NM_001081557, Exons 1–3, Mm.PT.58.15110314); and *Vamp3* (NM_009498, exons 1–3, Mm.PT.58.32746859). Primer sequences are available upon request or from the manufacturer (www.idtdna.com/site/order/qpcr/predesignedassay). Real-time 5× HOT FirePol qPCR master mix purchased from Mango Biotechnology was used to perform PCR reactions according to the manufacturer’s recommendations (Solis BioDyne) using a 7500 Fast Real-Time PCR system (Applied Biosystems). All qPCR reactions were quantified using the relative standard curve approach based on the manufacturer’s recommendations. Candidate genes *Camta1*, *Park7*, *Per3*, and *Vamp3* were normalized to β actin prior to determining B6:D2 expression ratios that exceeded a *P* value <0.05 assuming unequal variance between the populations.

### Statistics

Statistical analyses evaluating genetic interactions between GSTs by trial were carried out by either one-way ANOVA or Student’s *t*-tests using JMP version 10.0 (SAS Institute). Groups were defined for interaction studies between *Szs1* and *Esf1* based on their genotype at single nucleotide polymorphisms (SNPs) rs8259388 for *Szs1* and rs13478053 for *Esf1*. Correlational and eQTL mapping tools using BXD strains are freely available using GeneNetwork (http://www.genenetwork.org).

### Candidate gene selection for Esf1

Cis-eQTLs adjacent to *Esf1* were selected using the tools available on GeneNetwork. We initially used expression data from the UTHSC BXD Aged Hippocampus data generated from Affymetrix mouse gene 1.0 ST exon level arrays (dataset GN392, kindly made available by R. Williams) for correlational analyses with retest GST values. Additional datasets used to confirm these analyses included GN72 provided by Genome Explorations Inc. for the NIAAA as part of an SBIR grant to Dr. David Patel; GN46 that was generated by the UTHSC-SJCRH cerebellum transcriptome profiling consortium; and published datasets GN206 and GN281 (Overall *et al.* 2009; Mozhui *et al.* 2012). Candidate genes were chosen for correlational study based on their presence within a 2-Mb interval encompassing rs32163108 based on it having the highest significance for retest GST. Individual exons were interrogated for each gene in the surrounding 2-Mb interval using their relative expression among 68 of the 97 available BXD lines in this dataset. Expression values for individual exons from candidate loci were used in a mapping analysis with the PyLMM algorithm as implemented within GeneNetwork (Lippert *et al.* 2011). All exons having an SNP with a Likelihood Ratio Score (LRS) >10 within 20 Mb of rs32163108 were deemed to have *cis* effects for the expression change being interrogated. All genomic positions are listed relative to build GRCm38/mm10, except where noted. All exons showing differential expression were evaluated for SNPs that could impact hybridizations by interrogating the variant table for the respective gene using tools available at ensemble (http://www.ensembl.org) or the UTHSC Genome Browser (http://ucscbrowserbeta.genenetwork.org).

### Data availability

All seizure threshold data are available from the GeneNetwork website under accession numbers 18963–18981 (http://www.genenetwork.org/).

## Results

**Figure 1:**
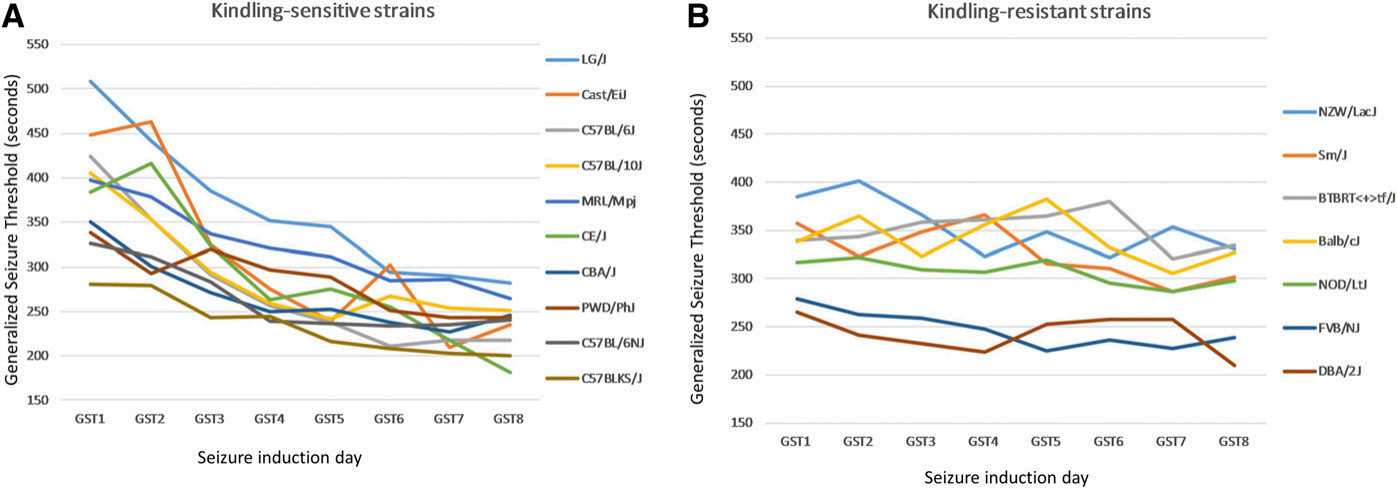
Inbred strain survey of GST over eight flurothyl-induced seizures. Latency to the loss of postural control [generalized seizure threshold (GST)] among several inbred strains of mice of diverse ancestry (*n* ≥ 6 per strain, see *Materials and Methods*). (A) Kindling-sensitive inbred strains were identified by calculating the slope of their daily GST (seconds) plotted against the induction phase seizure and selecting strains with a score <−10. (B) Kindling-resistant strains were classified by having a slope with the same score >−10. Note the clustering of kindling-resistant strains into two distinct groups (*P* < 0.001).

We previously reported that certain phenotypes quantified in the repeated-flurothyl seizure model are dissociable between B6, D2, and [B6×D2] F1 hybrids, suggesting that independent genetic factors mediated different aspects of seizure response (Papandrea *et al.* 2009a,b). Applying this repeated seizure model to a larger population of 17 inbred strains revealed three subgroups by plotting their daily GST response times over the eight induction trials (Figure 1). Most of the strains performed similar to B6 mice showing significant reductions in GST latency over the eight induction trials (*i.e.*, kindling-sensitive strains, Figure 1A) (Papandrea *et al.* 2009). Others behaved like D2 mice with statistically indistinguishable GST latencies over induction trials (Figure 1B). These kindling-resistant strains further clustered into two distinct groups (*P* < 0.0001), based on their average daily GST scores of 275 sec for the most sensitive groups (D2 and FVB mice) compared to 350 sec for the intermediate groups (BTBR, Balb, NZW, and Sm mice, Figure 1B).

We considered the possibility that the locus responsible for the separation in average GST between the kindling-resistant subgroups was due to *Szs1*; the strongest seizure susceptibility locus observed to date between B6 and D2 strains (Ferraro *et al.* 1997, 2004). However, haplotypes of kindling-resistant strains varied across the *Szs1* interval [as defined by Ferraro *et al.* (2004)] with no obvious region of correlation when these strains are aligned using the mouse phylogeny viewer [data not shown (Yang *et al.* 2011)]. Notably, the missense mutation reported to be responsible for *Szs1* effects in *Kcnj10* (Buono *et al.* 2004) was present in all kindling-resistant strains with the exception of BTBR. The clustering of kindling-resistant strains into two groups further supports a multigene model for genetic susceptibility during repeated-flurothyl-induced epileptogenesis and indicates additional uncharacterized modifiers other than *Szs1* are present in these strains.

### Repeated seizures shift genetic susceptibility over induction trials

We evaluated an additional 58 HMDP strains (Ghazalpour *et al.* 2012) with repeated seizures. Adding BXD recombinant inbred lines increased statistical power, while also providing access to publicly available expression data on GeneNetwork (Williams and Mulligan 2012). Our intent was to determine if previously mapped acute seizure susceptibility QTL, identified among B6, D2, or BXD mouse strains (Neumann and Collins 1991; Ferraro *et al.* 1997), were shared with flurothyl-induced seizures and to determine if repeatedly induced seizures influenced these genetic associations.

**Figure 2:**
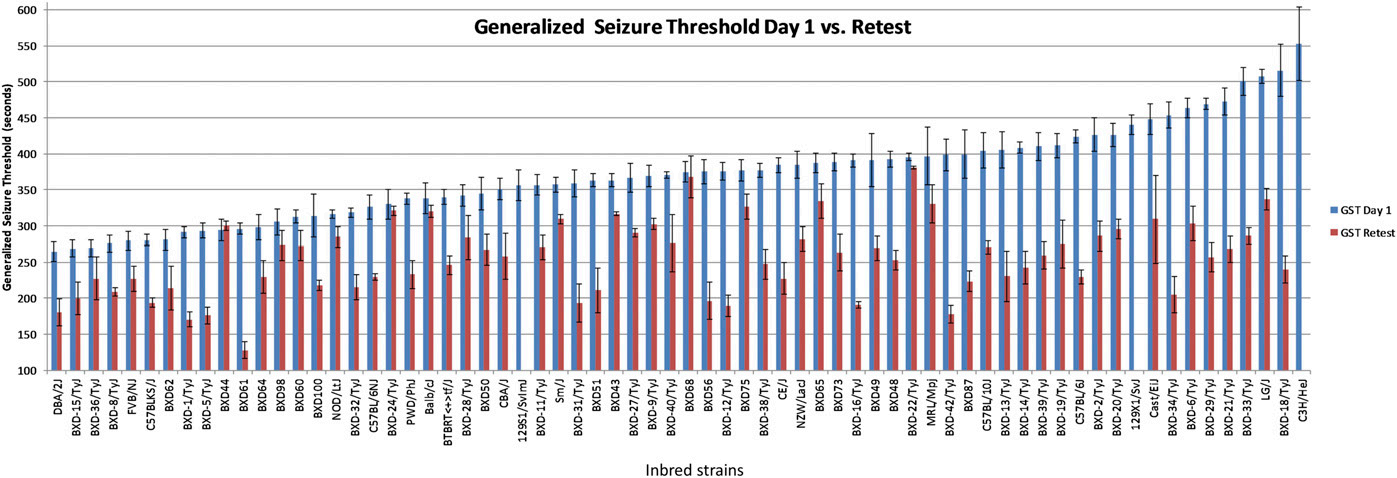
HMDP responses to initial and retest seizures spanning the repeated-flurothyl seizure model. Latency to loss of postural control (GST) for 68 HMDP strains surveyed for our association analyses. Blue bars indicate the initial GST (day 1) average for each strain with error bars showing the SEM. Red bars indicate retest GST averages for the strain with error bars showing the SEM. GST values are given in seconds.

There were diverse phenotypic responses across HMDP strains for initial and subsequent GSTs over seizure inductions (Figure 2 and Table 1). Mice exposed to the repeated-flurothyl seizure model had reduced interstrain variance upon retest compared to initial GST, but showed strong heritability at all seizure time points (*e.g.*, GST1 average: 375 sec; SD: 83.63 sec and retest GST average: 256 sec; SD 71 sec). We calculated the slope of induction phase GSTs (seizure trials 1–8) as a quantitative surrogate for the kindling effect seen previously (Samoriski and Applegate 1997). Kindling also had strong heritability and differences were highly significant (*P* < 0.0001) between strains (Table 1).

**Table 1:** GST values for HMDP strains over repeated-flurothyl seizures

Strain	GST1 Mean	GST1 SE	GST2 Mean	GST2 SE	GST3 Mean	GST3 SE	GST4 Mean	GST4 SE	GST5 Mean	GST5 SE	GST6 Mean	GST6 SE	GST7 Mean	GST7 SE	GST8 Mean	GST8 SE	GST Retest Mean	GST Retest SE	Kindling Mean	Kindling SE
129S1/SvImJ	356	21	410	10	425	28	379	18	368	23	335	29	312	11	364	5			−9	1
129X1/SvJ	440	14	406	20	394	21	405	50	325	43	298	39	368	12	394	48			−9	6
A/J	397	57	519	7	402	98	351	91	421	99	425	153	422	110	586	68	621	21	4	31
Balb/cJ	338	22	365	23	323	35	356	24	382	15	332	21	306	22	327	25	320	8	−5	3
BTBRT<+>tf/J	340	10	343	17	358	13	360	8	365	19	380	9	321	16	334	19	246	13	−1	3
BXD1/TyJ	291	8	283	11	243	10	210	7	204	9	195	8	192	14	183	12	170	11	−16	2
BXD100/RwwJ	314	29	300	11	282	7	274	11	241	15	240	11	214	18	205	17	218	7	−16	4
BXD11/TyJ	357	14	321	10	325	9	292	12	270	7	260	16	245	11	248	5	270	17	−17	2
BXD12/TyJ	375	12	289	8	281	9	257	8	228	9	187	5	238	8	220	10	189	15	−20	2
BXD13/TyJ	405	25	367	8	312	14	298	15	232	16	217	16	175	12	234	16	231	35	−30	2
BXD14/TyJ	409	7	384	18	275	10	311	18	247	7	277	15	255	12	234	22	243	22	−22	2
BXD15/TyJ	269	12	264	12	247	17	203	16	202	15	209	12	207	13	218	22	200	23	−9	2
BXD16/TyJ	391	9	307	9	298	14	258	13	253	13	238	9	230	16	224	12	191	4	−21	3
BXD18/TyJ	516	36	399	28	379	37	324	9	291	14	262	13	243	4	254	14	240	19	−36	3
BXD19/TyJ	411	17	423	50	327	30	306	10	320	36	299	22	265	30	314	38	275	33	−18	6
BXD2/TyJ	426	23	350	10	304	13	330	18	317	22	314	21	317	20	305	27	286	21	−9	3
BXD20/TyJ	426	16	330	18	292	18	289	20	281	20	273	16	262	13	267	19	296	13	−18	2
BXD21/TyJ	473	18	315	14	258	5	235	10	259	10	238	15	263	15	265	10	268	18	−21	2
BXD22/TyJ	395	5	370	7	331	5	346	12	254	36	339	19	326	7	291	11	381	2	−12	1
BXD24/TyJ	330	21	304	12	266	22	299	13	303	9	303	12	286	8	265	12	321	7	−5	3
BXD27/TyJ	366	20	361	15	326	8	320	12	304	12	310	15	284	9	285	9	290	6	−12	2
BXD28/TyJ	342	15	363	17	296	11	280	18	268	18	253	9	232	17	247	8	284	30	−17	2
BXD29/TyJ	469	8	394	19	352	19	331	9	325	13	290	28	260	24	270	22	257	20	−27	2
BXD31/TyJ	359	19	295	12	247	9	209	17	198	17	197	15	178	17	168	21	193	27	−25	4
BXD32/TyJ	318	7	265	11	268	9	288	15	265	17	259	6	257	9	256	20	216	17	−6	2
BXD33/TyJ	501	19	398	19	309	16	262	15	233	7	216	10	217	9	221	8	286	12	−38	3
BXD34/TyJ	454	18	285	16	258	3	229	12	220	11	219	12	234	13	233	13	205	25	−23	2
BXD36/TyJ	269	12	227	10	190	10	196	5	202	8	215	10	222	6	218	13	227	30	−4	2
BXD38/TyJ	377	10	338	10	268	10	234	8	217	6	219	15	193	18	172	11	247	21	−28	2
BXD39/TyJ	410	19	377	13	363	14	305	13	320	10	335	16	332	14	340	13	259	19	−9	2
BXD40/TyJ	371	5	324	10	262	9	236	17	179	14	193	18	212	20	199	16	276	40	−24	2
BXD42/TyJ	398	22	314	14	284	16	264	12	249	17	225	10	248	11	228	11	178	13	−20	3
BXD43/RwwJ	363	9	325	41	352	13	349	31	283	10	289	16	264	32	292	12	317	3	−13	3
BXD44/RwwJ	295	15	279	14	274	14	259	17	264	25	245	19	210	10	207	10	300	7	−12	2
BXD48/RwwJ	393	11	354	25	345	17	345	21	343	20	331	25	235	9	251	16	253	13	−19	3
BXD49/RwwJ	391	37	289	17	288	18	286	27	288	10	261	18	283	17	259	20	269	17	−12	3
BXD5/TyJ	293	10	263	14	243	11	222	5	242	11	229	8	229	11	226	15	176	12	−10	2
BXD50/RwwJ	345	23	308	20	214	18	243	9	252	10	256	18	248	23	236	12	268	21	−11	3
BXD51/RwwJ	363	9	309	16	265	39	220	24	207	25	200	1	228	15	254	23	211	31	−16	2
BXD55/RwwJ	422	99	388	23	208	16	246	40	208	19	218	7	188	12	191	5	334	111	−31	6
BXD56/RwwJ	375	17	345	28	312	25	297	19	198	30	301	25	170	40	243	21	196	26	−23	3
BXD6/TyJ	463	13	442	44	436	48	377	38	367	43	287	22	263	8	241	10	303	24	−16	5
BXD60/RwwJ	313	9	306	19	229	15	271	18	281	10	309	12	247	22	211	13	273	21	−9	2
BXD61/RwwJ	296	8	268	10	253	16	261	23	251	22	204	4	197	34	223	28	128	12	−12	2
BXD62/RwwJ	281	14	309	18	316	16	261	12	256	1	269	5	240	24	241	23	214	30	−9	4
BXD64/RwwJ	298	17	267	32	285	17	283	12	279	21	222	23	158	27	215	3	229	23	−16	4
BXD65/RwwJ	387	14	356	9	407	23	356	12	366	23	318	22	295	14	319	16	335	24	−12	1
BXD68/RwwJ	375	14	337	16	334	12	359	10	310	23	316	15	356	12	328	34	368	30	−4	4
BXD73/RwwJ	389	12	322	22	277	24	273	8	233	15	262	14	238	11	219	9	263	25	−20	3
BXD75/RwwJ	377	15	310	29	322	23	315	15	326	20	268	31	303	10	348	17	326	18	−5	4
BXD8/TyJ	276	12	250	14	207	14	229	22	194	12	188	13	188	14	178	7	209	5	−13	2
BXD87/RwwJ	400	34	287	12	288	33	220	15	229	14	220	19	212	10	200	11	224	14	−23	4
BXD9/TyJ	370	15	346	12	323	12	304	13	283	11	234	13	238	14	220	12	303	8	−21	2
BXD98/RwwJ	306	18	310	29	234	17	266	17	250	10	234	9	251	19	219	10	273	21	−11	2
C3H/HeJ	553	51	473	87	575		330		241		213		297		392				−35	
C57BL/10J	405	24	352	20	294	14	259	12	242	11	267	9	254	11	252	9	271	9	−19	3
C57BL/6J	424	9	353	9	291	6	256	6	238	10	211	6	217	9	218	7	229	10	−28	1
C57BL/6NJ	326	17	311	14	284	11	239	12	237	10	234	9	235	7	241	15	230	5	−13	3
C57BLKS/J	281	7	280	17	243	7	244	16	216	6	209	6	203	6	201	9	194	6	−13	1
Cast/EiJ	448	21	463	45	325	24	275	28	240	32	303	50	210	20	235	23	309	61	−34	4
CBA/J	351	15	301	15	272	19	250	16	252	16	238	15	227	17	245	16	258	32	−15	2
CE/J	384	10	416	22	323	17	263	14	275	13	255	33	217	17	182	19	228	22	−31	4
DBA/2J	265	13	241	11	233	9	224	12	252	12	258	17	257	14	210	19	180	19	−4	3
FVB/NJ	280	13	263	14	259	10	248	10	225	11	236	9	227	11	239	9	227	17	−7	3
LG/J	508	10	441	19	385	18	352	12	345	20	294	7	290	9	282	8	337	15	−32	2
MRL/Mpj	397	40	379	21	337	15	321	23	311	27	285	12	287	24	265	6	331	26	−18	3
NOD/LtJ	317	6	322	9	309	11	307	15	320	12	295	16	287	15	298	17	285	14	−6	3
NZW/LacJ	385	19	401	20	366	25	323	19	348	17	322	11	354	11	331	9	282	16	−9	2
PWD/PhJ	338	8	293	30	320	10	297	18	289	9	251	13	243	14	243	18	233	19	−16	4
Sm/J	357	10	324	20	348	8	367	15	316	11	310	10	286	9	302	11	309	6	−8	3
Average	373	18	337	19	304	17	286	17	271	18	263	17	252	16	257	16	262	20	−16	3
*h^2^*	0.55		0.71		0.54		0.75		0.70		0.79		0.62		0.36		0.50		0.94	

Approximately 4% of HMDP strains responded to flurothyl-induced seizures with high incidences of severe myoclonic seizures leading to brainstem seizures (Papandrea *et al.* 2009). These mice have pronounced jerking seizure incidents but do not lose postural control, which was our phenotypic criterion for GST onset. Such responses typically lead to increased flurothyl dosing while waiting for the animal to have a clonic seizure often leading directly to brainstem seizures (Papandrea *et al.* 2009). Any strains with >25% of animals exhibiting brainstem seizures on their 1st or 2nd trial were excluded from GWAS studies.

### Initial flurothyl-induced GSTs are consistent with previous models of seizure susceptibility

We used association analysis, correcting for genetic relatedness between strains with EMMA (Bennett *et al.* 2010). Daily GST response differences (in seconds) among HMDP mice were tested for association with 10^5^ SNPs having minor allele frequencies >0.05. Previous studies have established genome-wide significance for HMDP studies at 4.1 × 10^−6^ (Bennett *et al.* 2010).

**Figure 3:**
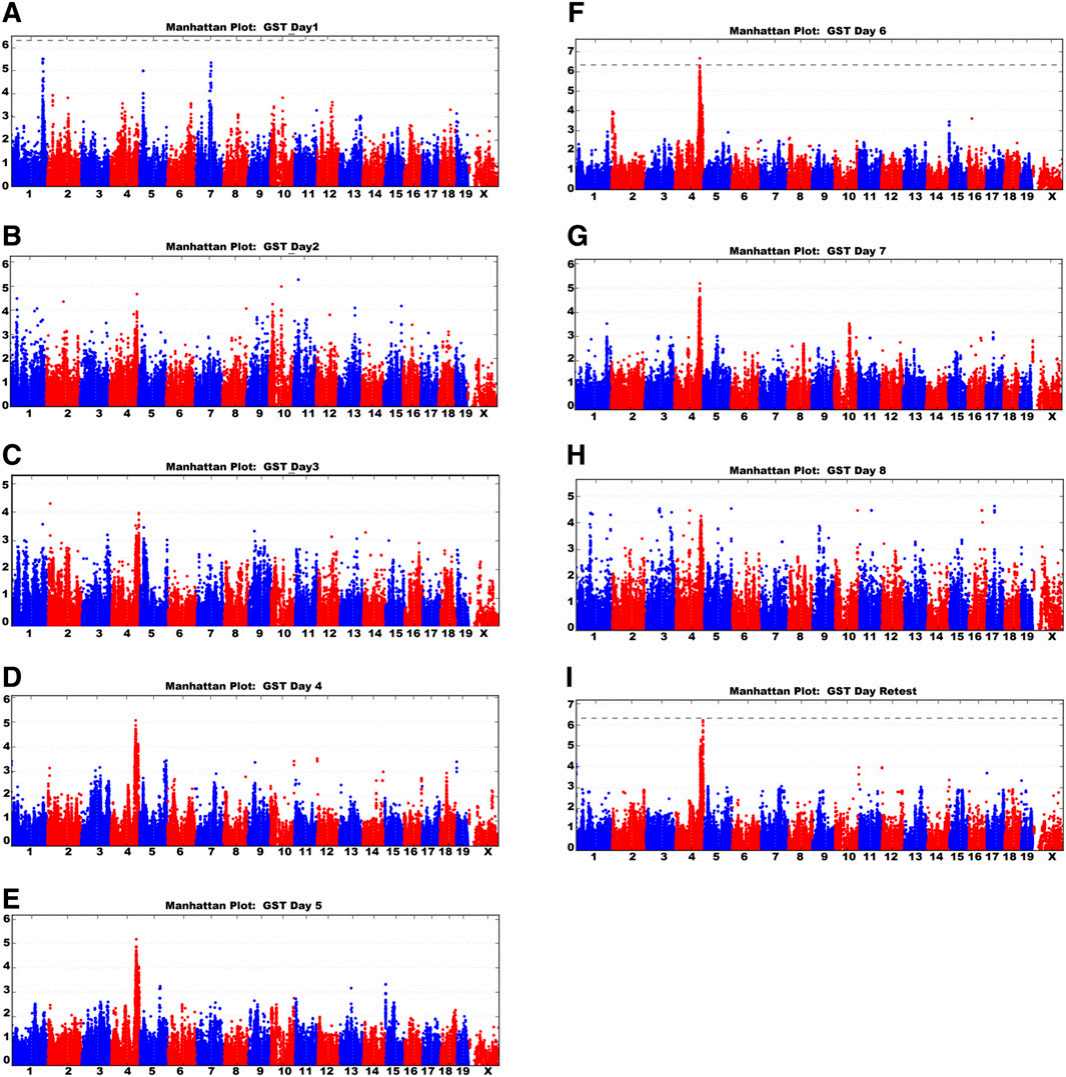
Genome-wide association results in the HMDP demonstrate a shift in seizure susceptibility over repeated-flurothyl-induced seizures. Manhattan plots for the seizure susceptibility associations over repeated seizures. (A) Acute seizure susceptibility associated with multiple suggestive associations >10^−4^ including chromosomes 1, 5, and 7. (B–E) Manhattan plots of day 2 to day 5 GST scores variance show a progression of associations to Distal Chromosome 4. (F) By the 6th induction seizure (day 6), associations exceeding genome-wide significance are detected on distal chromosome 4 near 139 Mb (GRCm38). (G and H) Manhattan plots of day 7 and day 8 GST variance. (I) Distal chromosome 4 associations persist until a retest seizure (given 4 wk after the induction period) with the five most significant SNP associations occurring in *Camta1* (151 Mb). Dotted line represents genome-wide significance (*P* < 4.4 × 10^−6^).

**Figure 4:**
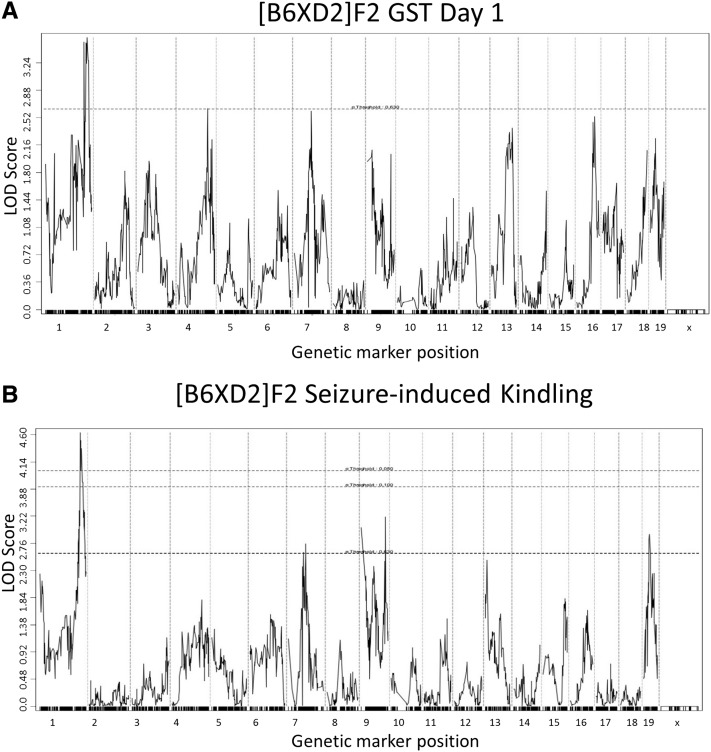
Regression analysis of [B6×D2] F2 intercross. (A) GST1 susceptibility was used as a quantitative trait to confirm the presence of a distal suggestive QTL corresponding to *Szs1*. (B) The slope of induction phase GSTs was used as a quantitative measure of kindling that also identified the *Szs1* locus.

Day 1 GST values among HMDP strains were associated with SNPs on mouse chromosome 1 that overlapped with the previously designated QTL *Szs1* (Ferraro *et al.* 2004; Chaix *et al.* 2007). However, this QTL, in addition to another on chromosome 5 near *Szs6* and a novel locus on mouse chromosome 7, did not reach the genome-wide significance cutoff (Figure 3 and Table 2). A [B6×D2] F2 intercross was also exposed to the repeated-flurothyl seizure model that was genotyped with the Mouse Universal Genotyping Array (Morgan *et al.* 2015). Multiple markers closely linked to the *Szs1* locus reached a suggestive genome-wide significance in this independent cross achieving a maximal LOD of 3.47 (Figure 4) confirming the similarities between our approach and alternative seizure studies.

**Table 2:** Top five SNP associations for kindling, GST1, GST6, and retest GST

Phenotype	Position (bp)*^a^*	−logP	SNP ID	M.A.F.*^b^*	Flanking Genes Within 500 kb of SNP*^c^*
Kindling	1:174420753	5.86E−09	rs3707910	0.1433	*Igfs8*, *Atp1a2*, ***Kcnj9***[Table-fn t2n3], ***Kcnj10***, *Pigm*, *Igsf9*, *Slamf9*, *Tafln2*, *Cfap45*, *Slamf8*, *Fcrl6*, *Dusp23*, *Crp*
	1:175151112	5.86E−09	rs8242481	0.182	*Olfr1408*, *Olfr1406*, *Olfr218*, *Mptx2*, *Olfr1404*, *Olfr418*, *Ackr1*, *Cadm3*, *Aim2*, *Gm4995*
	1: 174797513	6.06E−09	rs30463665	0.25	*Dusp23*, *Crp*, *Apcs*, *SNORA17*, *Olfr16*
	1: 176533310	1.40E−08	rs2020486	0.22	*Olfr417*, *Olfr248*, *Olfr414*, *olfr220*, *Fmn2*
	1: 174239958	1.41E−08	rs30472229	0.25	*Copa*, *Pex19*, *Dcaf8*, *Pea15a*, *Casaq1*, *Igfs8*, *Atp1a2*, ***Kcnj9***, ***Kcnj10***, *Pigm*
GST day 1	1: 174420753	3.15E−06	rs3707910	0.143	*Igfs8*, *Atp1a2*, ***Kcnj9***, ***Kcnj10***, *Pigm*, *Igsf9*, *Slamf9*, *Tagln2*, Cfap45, *Slamf8*, *Fcrl6*, *Dusp23*
	1: 175151112	3.15E−06	rs8242481	0.182	*Olfr16*, *Olfr1408*, *Olfr1406*, *Olfr218*, *Mptx2*, *Olfr1404*, *Olfr418*, *Ackr1*, *Cadm3*, *Aim2*
	1: 174455423	4.22E−06	rs31561177	0.4	*Igfs8*, *Atp1a2*, ***Kcnj9***, ***Kcnj10***, *Pigm*, *Igsf9*, *Slamf9*, *Tafln2*, *Cfap45*, *Slamf8*, *Fcrl6*, *Dusp23*, *Crp*
	7: 87409991	4.61E−06	rs4226715	0.2	*###Rik*, *Zfp710*, *idh2*, *Cib1*, *Ttll13*, *Ngrn*, *Rccd1*, *Prc1*, *Unc45a*, *Vps33b*, *Man2a2*, *Fes*, *Furin*, *Blm*
	1: 174239958	4.98E−06	rs30472229	0.25	*Ncstn*, *Copa*, *Pex19*, *Dcaf8*, *Pea15a*, *Casaq1*, *Igfs8*, *Atp1a2*, *Kcnj9*, *Kcnj10*, *Pigm*, *Igsf9*, *Slamf9*
GST day 6	4: 138646868	2.29E−07	rs32914632	0.2	*Otud3*, *Pla2g2a*, *Pla2g2e*, *Rnf186*, *Tmco4*, *Htr6*, *Nbl1*, *Minos*, *Capzb*, *Pqlc2*
	4: 138129729	5.93E−07	rs32007081	0.25	*Cda*, *Fam43b*, *Mul1*, *ABO41806*, ***Camk2n1***, *Vwa5b1*, *Ubxn10*, *Pla2g2c*, *Pla2g25*
	4: 137042287	6.80E−07	rs27577055	0.25	*Wnt4*, *Cdc42*, *Cela3a*, *Cela 3b*, *Hspg2*, *Ldlrad2*, *####Rik*, *Usp48*, *Rap1gap*
	4: 137353761	9.71E−07	rs8256572	0.33	*Ldlrad2*, *####Rik*, *Usp48*, *Rap1gap*, *Rap1gapcs*, *Alpl*, *Ece1*, *Eif4g3*
	4: 138291047	1.13E−06	rs27575102	0.4	*ABO41806*, ***Camk2n1***, *Vwa5b1*, *Ubxn10*, *Pla2g2c*, *pla2g25*, *Otud3*, *Pla2g2a*
GST retest	4: 150729074	6.53E−07	rs32163108	0.4	***Camta1***
	4: 150886138	7.71E−07	rs32757832	0.25	***Camta1***
	4: 150659779	1.11E−06	rs13478053	0.5	*Vamp3*, ***Camta1***
	4: 150681054	1.97E−06	rs32590839	0.667	*Uts2*, *Per3*, *Vamp3*, ***Camta1***
	4: 150745317	2.26E−06	rs32180816	0.5	***Camta1***

a Position relative to NCBI Build 37 genome assembly.

b Minor allele frequency.

c Genes in bold are considered the best candidates for influence over trait.

GWAS analysis performed with day 2 GST data greatly diminished significance associations across the genome causing drops in associations observed on the previous day (Figure 3). By day 3, the genome-wide level of significant associations continued to decline compared to day 1 GST associations, as novel SNP associations increased on distal chromosome 4. The nonsignificant chromosome 4 associations persisted in subsequent induction phase GST Manhattan plots (Figure 3) achieving genome-wide significance on day 6 of the induction phase (Figure 3).

We hypothesized that QTL effects over the eight induction trials were dynamic because of homeostatic plasticity responses to induced seizures (Turrigiano and Nelson 2004). Repeated seizures exacerbated environmental variance limiting our ability to detect significant QTL in early induction trials. As the CNS adapted to the heightened state of excitability that was elicited by the induction phase seizures, a window of significant associations was revealed in the latter half of this period.

### Seizure-induced kindling effects are also associated with Szs1

**Figure 5:**
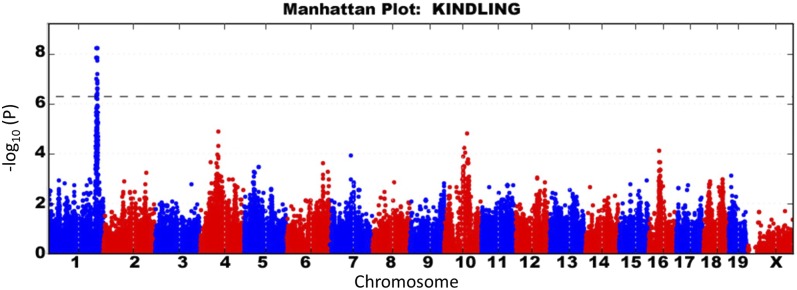
Genome-wide association results in the HMDP for flurothyl kindling. Using the slope of induction phase GSTs as a measure of kindling, distal chromosome 1 associations, overlapping with *Szs1*, were identified as the strongest genetic effect. Dotted line represents genome-wide significance (*P* < 4.4 × 10^−6^).

To better understand the genetic basis for the seizure-induced kindling phenomenon, we calculated the slope of induction phase GSTs over 8 d to evaluate kindling effects as a quantitative trait (Table 1). In this manner, we hoped to minimize day to day environmental influence on seizure susceptibility while enhancing the genetic effects in seizure-induced brain plasticity. Association analysis of the kindling effect revealed SNPs that once again overlapped with the *Szs1* locus (Figure 5 and Table 2), but this time exceeding genome-wide significance (maximum −log p 5.86 e−09). The effects of *Szs1* on kindling were also confirmed in our [B6×D2] F2 cross, achieving a maximum genome-wide significance of *P* < 0.05 at a LOD of 4.60 (Figure 4).

### Completion of the repeated-flurothyl seizure model reveals novel associations on chromosome 4

Like flurothyl, repeated doses of kainic acid induce both short-term and long-term effects on epileptogenesis in mice (Tse *et al.* 2014). We have demonstrated that eight flurothyl-induced generalized seizures led to a transient spontaneous seizure state similar to kainic acid epileptogenesis (Kadiyala *et al.* 2016), but unlike kainic acid, flurothyl induces no obvious neuronal death and is a generalized seizure model (Kadiyala *et al.* 2016; Kadiyala and Ferland 2017). To understand how such generalized spontaneous seizures could further influence genetic susceptibility, we left flurothyl-exposed mice undisturbed for 28 d, retesting their seizure thresholds with an additional flurothyl-induced seizure on the final day (*i.e.*, retest seizure). As shown in Figure 3, retest GST was associated with significant SNP associations in the distal portion of chromosome 4, with the top five SNPs residing within the *Calmodulin Binding Transcription Activator 1* (*Camta1*) gene (Figure 3 and Table 2).

Chromosome 4 GWAS associations were detected at two distinct time points and locations during the repeated-flurothyl seizure model. We first detected a locus with maximal effect (−log p 2.29 e−07) at rs32914632 near 138 Mb on chromosome 4 in the 6-d GST data (Figure 3 and Table 2), while retest GST had associations distal to this region that were highest (−log p 6.53 e−07) at rs32163108 near 150 Mb on chromosome 4 (Figure 3 and Table 2). While the day 6 association may overlap with the previously identified β-carboline-induced seizure QTL *Bis1*, congenic analyses performed by others in conjunction with our mapping results exclude the retest association from overlap with this locus (Zerr *et al.* 2000; B. Martin, personal communication). Based on this information, we designate the retest GST association as *Epileptogenesis susceptibility factor 1* (*Esf1*); a novel seizure susceptibility locus that is dependent on prior seizure exposure to manifest its effects.

### The D2 allele of the Szs1 locus influences Esf1 effects

**Figure 6:**
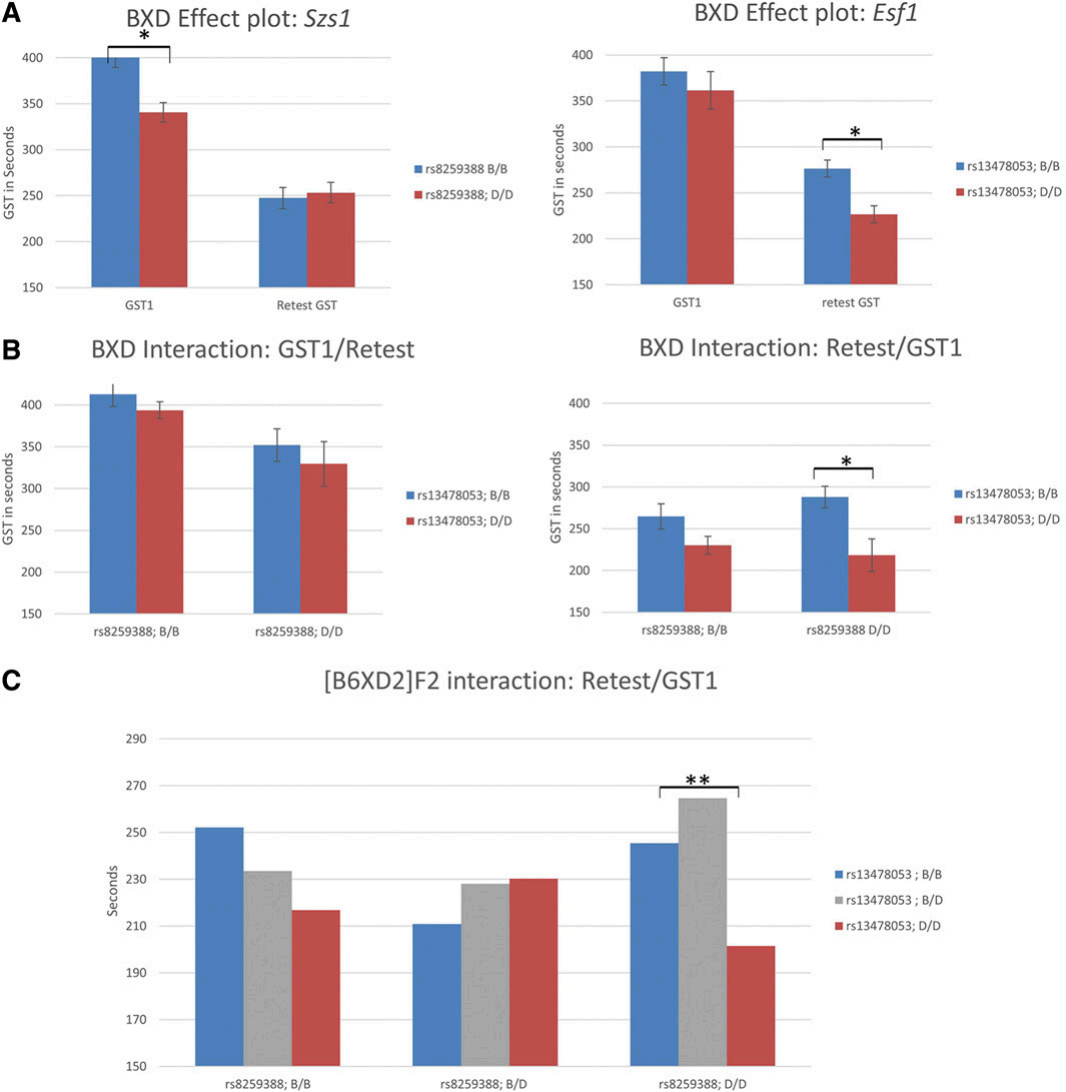
Interaction between *Szs1*/GST1 and *Esf1*/retest GST. (A) BXD strains were sorted according to their genotype for rs8259388 within the *Szs1* locus and rs13478053 within *Esf1* to compare their effects on their respective phenotypes. Significant differences in response between the groups are indicated by an asterisk. (B) The *Szs1* subgroups were further divided according to their *Esf1* genotype to compare the average retest GST in these four groups. Significant differences (*P* < 0.05 marked by an asterisk) in retest GST were seen between B6 and D2 mice carrying the D2 allele of *Szs1*, but not the B6 allele. (C) Similar sorting for the two loci was performed in a [B6×D2] F2 intercross to reveal a similar pattern of significance between B6 and D2 homozygous animals, but not heterozygotes.

We considered the possibility that the temporal position of *Szs1* and *Esf1* effects in our model may indicate an interaction between these loci driven by repeated seizures. We tested for interdependency between the two genetic states by segregating BXD strains based on their genotype at *Szs1* (*i.e.*, B6 or D2 allele at rs8259388) to compare respective GSTs at either end of the seizure protocol (GST1 and retest GST). As expected, the average response between B6 and D2 *Szs1* groupings was significantly different for GST1 (*P* = 0.001), but not for retest GST (Figure 6). Conversely, comparisons using *Esf1* SNP rs13478053 to sort B6 *vs.* D2 alleles in BXDs showed significant differences in retest GST (*P* < 0.001), but not GST1 response. Notably, when the B6 and D2 *Szs1* subsets were further divided based on their genotypes at *Esf1*, we only observed significant differences between the D2-*Szs1*/B6-*Esf1* compared to the D2-*Szs1*/D2-*Esf1* subset (Figure 6). One possible explanation for this outcome is that the D2-*Szs1* haplotype potentiates the effects of *Esf1* in mice undergoing repeated seizures. These effects were also observed in our [B6×D2] F2 cohort (Figure 6C) confirming the dependencies between the opposite ends of our seizure paradigm.

### Genes tightly linked to Esf1 are influenced by cis-eQTL in BXD mice

Legacy data generated on several collections of recombinant inbred lines and genetic reference populations are available on the GeneNetwork systems genetics resource website (Williams and Mulligan 2012). Among these data, RNA expression profiles have compared gene expression levels between BXD strains for several brains regions (Chesler *et al.* 2005). Mapping expression differences as quantitative traits can reveal genetically controlled expression QTL (eQTL), a subset of which map to the same location as the differentially expressed gene (*i.e.*, *cis*-eQTL) (Mortazavi *et al.* 2008; Wang *et al.* 2012). We exploited this resource as a first-pass evaluation of candidate loci in the vicinity of *Esf1*.

**Figure 7:**
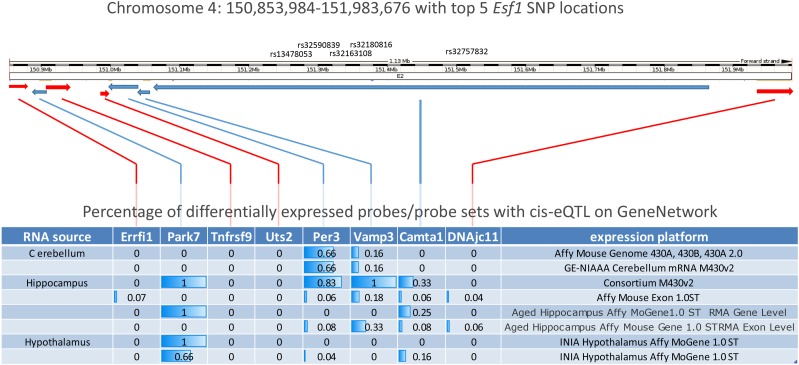
Summary figure of *Esf1* candidate loci *cis*-eQTL. Schematic of the genomic interval surrounding *Esf1* including the approximate location of top SNPs from GWAS analysis (top) and the relative size and orientation of genes in the vicinity (red arrows indicate plus strand, blue arrows indicate minus strand) adapted from the Ensembl website (GRCm38). Table showing percentage of probes sets showing *cis*-eQTL/candidate gene from various datasets available on the GeneNetwork systems genetics resource. Note predominance of *cis*-eQTL on genes on the minus strand.

We interrogated mouse hippocampus expression data available on GeneNetwork comparing the relative expression among BXD strains subjected to the repeated-flurothyl seizure model. Expression differences between BXD strains were observed in genes across the *Esf1* interval (Table 3). Among these expressed differences, multiple *cis*-eQTLs were detected. A cluster of probe sets with highly significant LRS (>25) were detected within four genes: *Camta1*, *Period 3* (*Per 3*), *Parkinson protein 7* (*Park 7*), and *Vesicle Associated Membrane Protein 3* (*Vamp3*) (Figure 7 and Table 3). These *cis*-eQTLs attained the highest significance in the distal portion of the *Camta1* gene directly adjacent to the region with our top SNP associations for *Esf1* (Figure 7).

**Table 3:** eQTL whose expression correlates with retest GST around *Esf1*

SNP ID	Position*^a^*	Transcript Influenced*^b^*	LRS	R2*^c^*
D4Mit343	142711337	Vamp3(3)	21	−0.21
rs13478031	144528745	Errfi1(1)	14	0.03
rs3669806	148822719	Nol9(5)	9	**−0.15**
rs3697097	150397103	Per3 (2)	30	**0.38**
rs3697097	150397103	Camta1(7)	52	**0.61**
rs3697097	150397103	Camta1(10)	72	**0.64**
rs4140148	150476099	Park7(2)	46	**0.26**
rs6358921	150476099	Vamp3(3)	21	−0.21
rs6358921	150476099	Vamp3(4)	28	**−0.31**
rs3680006	151497797	Tnfrsf25(7,8)	10	0.18
rs13478063	153325361	Camta1(9)	10	**0.37**
rs32463773	156010835	Camta1(13)	13	0.22
rs6279100	156183528	Per3(6)	10	−0.07

a Position relative to NCBI Build 38 genome assembly.

b Gene symbol (exon containing probe/probe set).

c Correlations with retest GST in bold *P* < 0.05; underlined bold *P* < 0.0001.

To better define the relationship between these *cis*-eQTL and *Esf1*, we correlated the expression values for each probe set with retest GST values. Most probe sets with LRS over 25 were significantly correlated (*P* < 0.05) with retest GST (Table 3). Exons from *Vamp3* showed negative correlations with *Esf1*, while those for *Camta1*, *Per3*, and *Park7* had positive correlations (Table 3). The most significant correlations (*P* < 0.0001) were observed in two *Camta1* probes present in exons 7 and 10 of *Camta1* (Table 3).

Similar *cis*-eQTLs were found in other expression datasets using distinct arrays and brain tissues (Figure 7). The percentage of probes within candidate genes showing *cis*-eQTL (selected based on a LRS score of 15 or greater within 2 Mb of a given locus) trended toward tissue-specific patterns. For example, while *Camta1* and *Park7* had *cis*-eQTL in hypothalamus (Figure 7, Geo accession GSE36674), *Vamp3* and *Per3* eQTL were more likely to be detected in cerebellum. Hippocampal *cis*-eQTLs were detected for all four loci in the region, further supporting a locally controlled difference in transcription that is differentially regulated between B6 and D2 animals.

We confirmed the presence of differential expression between B6 and D2 inbred strains using qPCR. We compared control cerebellum and hippocampus from age-matched B6 and D2 animals as well as similar sets that had undergone the full flurothyl induction protocol, collecting tissue 90 min after the last seizure. As shown in Table 4, significant differences in expression (*P* < 0.05) were observed between B6 and D2 hippocampus in the seizure-naive set for *Camta1*, *Park7*, and *Per3*, but not in cerebellar mRNA. In mice undergoing repeated-flurothyl seizures, there was an induction of *Park7* and *Vamp3* in B6 cerebellum that did not occur in D2 cerebellum. However, only *Camta1* expression in hippocampus was significantly and differentially expressed between B6 and D2 mice after seizures.

**Table 4:** Quantitative PCR of expression levels in *cis*-eQTL genes in B6 and D2 brain tissues

		Preseizure Exposure			Postseizure Exposure		
Tissue	Gene	B6/D2 Ratio	Expression B6/D2*^a^*	*P* value	B6/D2 Ratio	Expression B6/D2*^a^*	*P* value
Cerebellum	Camta1	0.82	0.92(0.1)/1.18(1.1)	0.9508	0.83	1.0(0.14)/1.2(0.17)	0.7964
**Hippocampus***^b^*	**Camta1**	**2.08**	**0.54(0.07)/0.28(0.02)**	**0.0019**	**1.96**	**0.49(0.34)/0.25(0.08)**	**0.0434**
Cerebellum	Park7	0.53	0.33(0.03)/0.62(0.16)	0.9246	1.31	0.67(0.11)/0.51(0.19)	0.1243
**Hippocampus**	**Park7**	**1.63**	**0.36(0.06)/0.19(0.03)**	**0.0317**	1.84	0.31(0.23)/0.53(0.28)	0.0903
Cerebellum	Per3	1.32	1.25(0.19)/0.95(0.12)	0.1172	1.14	1.04(0.25)/0.91(0.16)	0.344
**Hippocampus**	**Per3**	**2.10**	**0.86(0.15)/0.41(0.07)**	**0.0196**	0.55	0.6(0.07)/0.54(0.19)	0.9797
Cerebellum	Vamp3	0.46	0.51(0.02)/1.23(0.43)	0.9051	1.40	1.25(0.37)/0.89(0.47)	0.1967
Hippocampus	Vamp3	1.33	0.36(0.03)/0.27(0.06)	0.1632	0.58	0.38(0.04)/0.66(0.15)	0.93

a Expression levels quantified using relative standard curve approach normalized to β actin mRNA levels (SEM).

b Data listed in bold indicates *P* < 0.05.

## Discussion

### The repeated-flurothyl seizure model is a novel approach to experimental epileptogenesis

This work demonstrates a novel preclinical approach to discover gene by environment interactions that mediate epileptogenesis. While rodent genetic studies have uncovered important seizure mechanisms among mammals (Hall 1947; Fletcher *et al.* 1996; Buono *et al.* 2004; Ferraro *et al.* 2004), ours is the first to follow genetic susceptibility over repeated seizures. Thus, unique modifiers dependent upon prior seizure activity can be discovered that are risk indicators or potential treatment targets for epilepsy.

A detailed genetic analysis of the *Szs1* QTL locus has given insight into the molecular basis of seizure susceptibility (Ferraro *et al.* 2004). Congenic and transgenic rescue studies in mice have led to the election of a coding mutation within *Kcnj10* as the strongest candidate for *Szs1* differences in maximal electroshock threshold (Ferraro *et al.* 2004). *KCNJ10* associations are also observed in individuals with epilepsy (Buono *et al.* 2004; Lenzen *et al.* 2005), indicating that similar epilepsy mechanisms are shared between rodents and humans. Additional work has shown that *Kcnj10* function in glia is critical in K^+^ clearance, leading to increased neuronal hyper-excitability when conditionally deleted from astrocytes (Djukic *et al.* 2007). We show here that flurothyl-induced seizures are also influenced by *Szs1*, indicating a common connection between our approach and other experimental epilepsy models.

### Dynamic QTL effects are observed across multiple flurothyl-induced seizures

While association of the *Szs1* locus with day 1 GST was confirmed, *Szs1* associations were not detected on subsequent seizure trials. Using the slope of daily GST reductions over eight trials as a measure of kindling, we detected highly significant associations within *Szs1*, 100 kb distal to *Kcnj10*. We are now investigating how seizure-dependent factor(s) could influence *Szs1* effects, either by suppression of an existing *Szs1* mutation, like the reported *Kcnj10* mutation, or via alterations in other genes in this region. Multiple QTL affecting a variety of neurological traits in mice are present on distal chromosome 1 (Mozhui *et al.* 2008) that could be responsible for transient *Szs1* effects on seizure susceptibility.

Reductions in astroglial Kir4.1 channels (formed by *Kcnj10*) have been observed in both human temporal lobe epilepsy and experimental epilepsy models (Schroder *et al.* 2000; Losi *et al.* 2012; Steinhäuser *et al.* 2012). Thus, the negative correlation between day 1 GST and kindling (*r*^2^ = −0.71) could be due to suppression of *Kcnj10* activity in subsequent seizure trials. Reductions in *Kcnj10* activity may be a common feature of repeated seizures that are mediated by uncharacterized mechanisms of epileptogenesis. In our model, suppressing both normal and mutant alleles of *Kcnj10* as a means of seizure adaptation would consequently remove the *Szs1* effects from associations after the initial seizure. The mechanisms responsible for these effects may be within the *Szs1* interval or could be *trans*-acting factors that modify the effects of *Szs1*. While environmental confounds like animal age could contribute to seizure progression, all animals in our study were 6–7 wk old at the onset of the analysis. The presence of significantly different rates of kindling among HMDP strains suggests genetic factors mediate this activity within the HMDP. However, poor correlation between HMDP strain haplotypes within *Szs1* and kindling in our data indicates that the uncharacterized kindling modifier and *Szs1* are likely unlinked.

### Novel locus on distal chromosome 4 influences seizures after repeated seizures

As repeated seizures are administered, seizure-related brain responses are invoked to stabilize neuronal function (González *et al.* 2015). Thus, detection of significant QTL effects are likely confounded by increased environmental variance during induction phase seizures after the initial seizure. Kindling-sensitive strains, like B6, show GST values that stabilize toward the second half of the induction phase (Papandrea *et al.* 2009). Once all strains reach this plateau, a second window of heritable effects can be revealed.

Significant SNP associations reappear on day 6 in the induction phases that correspond with the timing of the GST plateau effect seen in B6 mice (Papandrea *et al.* 2009). Among the potential candidates for day 6 GST associations is the *calcium/calmodulin-dependent protein kinase II inhibitor* (*Camk2n*), whose inhibition is reported to result in epileptiform activity (Murray *et al.* 2000) making it a viable candidate gene for modifying epileptogenesis. Also, calcium signaling is an important factor in epilepsy progression (Pal *et al.* 2001). Ongoing work will establish the parameters that influence such transient susceptibilities and their relationship to seizure progression.

Seizure-driven epileptogenesis is predicted to continue into the incubation phase of the repeated-flurothyl model due to a period of spontaneous seizures where B6 mice have as many as six seizures a day remitting to less than one seizure a day by the end of our model (Kadiyala *et al.* 2016). Interestingly, D2 mice also develop spontaneous seizures following flurothyl induction, however at lower frequencies than B6 mice, but that persist throughout the 28-day incubation period (Kadiyala and Ferland, 2017). When retest seizures are delivered, *Esf1* is the predominant effect controlling seizure susceptibility with the top five associations all occurring in *Camta1*.

### Genotype of Szs1 can influence the effects of Esf1

Complex networks of neuronal adaptation are activated by seizures (Kadiyala *et al.* 2015) that are further influenced by genetic background, leading to differential outcomes in epilepsy development. Based on our data, mutations in *Kcnj10* segregating in the HMDP are associated with initial seizure effects that diminish over time, which may also be true in humans with epilepsy, raising the question of the nature of the association of *Kcnj10* with epilepsy. A possibility is that the effect of *Kcnj10* deficiency on seizure susceptibility is twofold. Its primary effect on hyper-excitability is mitigated by repeated seizures, but its effects on baseline K^+^ buffering in the interictal period remains abnormal. Such imbalances in K^+^ would be amplified in the context of repeated seizures leading to imbalances in downstream signaling cascades, in particular, Ca^2+^ signaling that has been shown to be altered by the presence of functional Kir4.1 channels (Härtel *et al.* 2007).

We show that strains carrying D2 alleles of *Szs1*, which is likely due in part to deficits in *Kcnj10*, has a significant impact on retest GST when these mice are further segregated based on their *Esf1* genotype, while those carrying a B6 *Szs1* haplotype have reduced effect on *Esf1* outcome. Thus, differences in *Szs1* could prime the ultimate outcomes that are observed in *Esf1*, possibly through an imbalance in steady-state K^+^ levels that exacerbate alternative mechanisms of epileptogenesis either within astrocytes or through effects on neuronal networks.

### System genetics analyses point to coordinate deregulation between B6 and D2 genes near *Esf1*

Utilization of BXD strains was important to our project in two ways. First, it added increased power in our association analyses that would not have been possible with inbred strains alone (Bennett *et al.* 2010). The BXD strains also have a unique advantage in legacy data that is publicly available to perform correlation and system genetic studies to further characterize the genetic basis of epileptogenesis (Williams and Mulligan 2012). The presence of *cis*-eQTL around *Esf1* was previously associated with other neuronal complex traits, including stress response (Wang *et al.* 2012). The effects were primarily attributed to differential expression of *Per3*. We also found differential baseline expression in *Per3* in B6 and D2 mice (Table 4), depending on the tissue type being interrogated, but this differential expression was not observed in mice completing the repeated-flurothyl model and did not correlate as well with retest GST data as *Camta1* expression differences.

Confirmation of the differential expression of *cis*-eQTL adjacent to *Esf1* was also observed in the hippocampus of B6 and D2 parental strains by qPCR. While three of the four candidate loci showed significant differences in mRNA prior to seizures, only *Camta1* maintained significant differences after completion of the repeated-flurothyl model. The differential expression of these loci was consistently higher in B6 mice indicating that a global regulatory control may impact this effect. Thus, our observation that the two SNPs rs369709 and rs3697097 that account for all of the *cis*-eQTL with LRS over 20 map to a region proximal to *Esf1* could provide a good place to compare these genomes for transcriptional elements that may facilitate this kind of coordinated differential transcription.

Each of these potential candidates for *Esf1* have plausible ways they could contribute to epileptogenesis. As mentioned above, *Per3* has previously been implicated in neuronal stress response and other gene family members have been implicated in induced seizures in mice (Gachon *et al.* 2004). *Park 7* protects against oxidative stress and may protect neurons from death (Kolisek *et al.* 2015) and has also been shown to be overexpressed in hippocampi of individuals with epilepsy (Persike *et al.* 2012). *Vamp3* is a downstream target of MeCP2 that mediates SNARE-dependent exocytosis of glutamate (Kandratavicius *et al.* 2014). While these genes have reduced significance, based on our GWAS associations and system genetics data, they will need to be considered for their potential contributions to epileptogenesis going forward.

*Camta1* is an excellent candidate for epileptogenesis susceptibility. In addition to having the five most significant SNPs associated with retest GST, and the best correlation with eQTLs in our system genetics data, it has several known functions that make it a good candidate from a biological perspective. First, calmodulin is a well-known mediator of postictal brain activity and has been implicated in brain adaptation to chemical (DeLorenzo 1986) and electrical kindling (Goldenring *et al.* 1986). Camta proteins are well-known mediators of the stress response in multiple species (Pandey *et al.* 2014; Doherty *et al.* 2009) and are well suited to mediate the stress response from repeated seizures. Increased Camta1 activity is a predictable consequence of inducing repeated seizures, the activity of which would be enhanced by reduced K^+^ buffering capacity that is an aspect of our model. Finally, mutations in *CAMTA1* are associated with multiple neurological phenotypes, including seizure susceptibility in humans (Miller *et al.* 2011; Shinawi *et al.* 2015). Identifying the basis of this susceptibility will lead to improved diagnosis and novel treatment options for individuals with epilepsy.

## References

[bib1] Bennett B. J. Farber C. R. Orozco L. Kang H. M. Ghazalpour A., 2010 A high-resolution association mapping panel for the dissection of complex traits in mice. Genome Res. 20: 281–290. 20054062 10.1101/gr.099234.109 PMC2813484

[bib2] Broman K. W. Wu H. Sen S. Churchill G. A., 2003 R/qtl: QTL mapping in experimental crosses. Bioinformatics 19: 889–890. 12724300 10.1093/bioinformatics/btg112

[bib3] Buono R. J. Lohoff F. W. Sander T. Sperling M. R. O’Connor M. J., 2004 Association between variation in the human KCNJ10 potassium ion channel gene and seizure susceptibility. Epilepsy Res. 58: 175–183. 15120748 10.1016/j.eplepsyres.2004.02.003

[bib4] Chaix Y. Ferraro T. N. Lapouble E. Martin B., 2007 Chemoconvulsant-induced seizure susceptibility: toward a common genetic basis? Epilepsia 48: 48–52. 17910581 10.1111/j.1528-1167.2007.01289.x

[bib5] Chesler E. J. Lu L. Shou S. Qu Y. Gu J., 2005 Complex trait analysis of gene expression uncovers polygenic and pleiotropic networks that modulate nervous system function. Nat. Genet. 37: 233–242. 15711545 10.1038/ng1518

[bib6] DeLorenzo R. J., 1986 A molecular approach to the calcium signal in brain: relationship to synaptic modulation and seizure discharge. Adv. Neurol. 44: 435–464. 3010680

[bib7] Djukic B. Casper K. B. Philpot B. D. Chin L.-S. McCarthy K. D., 2007 Conditional knock-out of Kir4.1 leads to glial membrane depolarization, inhibition of potassium and glutamate uptake, and enhanced short-term synaptic potentiation. J. Neurosci. 27: 11354–11365. 17942730 10.1523/JNEUROSCI.0723-07.2007 PMC6673037

[bib8] Doherty C. J. Van Buskirk H. A. Myers S. J. Thomashow M. F., 2009 Roles for Arabidopsis CAMTA transcription factors in cold-regulated gene expression and freezing tolerance. Plant Cell 21: 972–984. 19270186 10.1105/tpc.108.063958 PMC2671710

[bib9] England M. J. Liverman C. T. Schultz A. M. Strawbridge L. M., 2012 Epilepsy across the spectrum: promoting health and understanding: a summary of the Institute of Medicine report. Epilepsy Behav. 25: 266–276. 23041175 10.1016/j.yebeh.2012.06.016 PMC3548323

[bib10] Engstrom F. L. Woodbury D. M., 1988 Seizure susceptibility in DBA and C57 mice: the effects of various convulsants. Epilepsia 29: 389–395. 3292231 10.1111/j.1528-1157.1988.tb03736.x

[bib54] Ferland, R. J. 2017 The repeated flurothyl seizure model in mice. Bio-protocol 7(11): e2309. DOI: 10.21769/BioProtoc.2309. 10.21769/BioProtoc.2309 PMC5524139 28748202

[bib11] Ferraro T. N., 2016 Barriers to the use of genetic information for the development of new epilepsy treatments. Expert Rev. Neurother. 16: 5–8. 26559170 10.1586/14737175.2016.1115718

[bib12] Ferraro T. N. Golden G. T. Smith G. G. Schork N. J. St. Jean P., 1997 Mapping murine loci for seizure response to kainic acid. Mamm. Genome 8: 200–208. 9069121 10.1007/s003359900389

[bib13] Ferraro T. N. Golden G. T. Smith G. G. Martin J. F. Lohoff F. W., 2004 Fine mapping of a seizure susceptibility locus on mouse Chromosome 1: nomination of Kcnj10 as a causative gene. Mamm. Genome 15: 239–251. 15112102 10.1007/s00335-003-2270-3

[bib14] Fletcher C. F. Lutz C. M. O’Sullivan T. N. Shaughnessy J. D. Hawkes R., 1996 Absence epilepsy in tottering mutant mice is associated with calcium channel defects. Cell 87: 607–617. 8929530 10.1016/s0092-8674(00)81381-1

[bib15] Gachon F. Fonjallaz P. Damiola F. Gos P. Kodama T., 2004 The loss of circadian PAR bZip transcription factors results in epilepsy. Genes Dev. 18: 1397–1412. 15175240 10.1101/gad.301404 PMC423191

[bib16] Ghazalpour A. Rau C. D. Farber C. R. Bennett B. J. Orozco L. D., 2012 Hybrid mouse diversity panel: a panel of inbred mouse strains suitable for analysis of complex genetic traits. Mamm. Genome 23: 680–692. 22892838 10.1007/s00335-012-9411-5 PMC3586763

[bib17] Goldenring J. R. Wasterlain C. G. Beate Oestreicher A. de Graan P. N. E. Farber D. B., 1986 Kindling induces a long-lasting change in the activity of a hippocampal membrane calmodulin-dependent protein kinase system. Brain Res. 377: 47–53. 3730855 10.1016/0006-8993(86)91189-3

[bib18] González O. C. Krishnan G. P. Chauvette S. Timofeev I. Sejnowski T., 2015 Modeling of age-dependent epileptogenesis by differential homeostatic synaptic scaling. J. Neurosci. 35: 13448–13462. 26424890 10.1523/JNEUROSCI.5038-14.2015 PMC4588612

[bib19] Grone B. P. Baraban S. C., 2015 Animal models in epilepsy research: legacies and new directions. Nat. Neurosci. 18: 339–343. 25710835 10.1038/nn.3934

[bib20] Gupta S. Kwan P. Faught E. Tsong W. Forsythe A., 2016 Understanding the burden of idiopathic generalized epilepsy in the United States, Europe, and Brazil: an analysis from the National Health and Wellness Survey. Epilepsy Behav. 55: 146–156. 26773686 10.1016/j.yebeh.2015.12.018

[bib21] Hall C. S., 1947 Genetic differences in fatal audiogenic seizures between two inbred strains of house mice. J. Hered. 38: 2–6. 20289559

[bib22] Härtel K. Singaravelu K. Kaiser M. Neusch C. Hülsmann S., 2007 Calcium influx mediated by the inwardly rectifying K+ channel Kir4.1 (KCNJ10) at low external K+ concentration. Cell Calcium 42: 271–280. 17284334 10.1016/j.ceca.2006.12.004

[bib23] International League Against Epilepsy Consortium on Complex Epilepsies, 2014 Genetic determinants of common epilepsies: a meta-analysis of genome-wide association studies. Lancet Neurol. 13: 893–903. 25087078 10.1016/S1474-4422(14)70171-1 PMC4189926

[bib24] Kadiyala S. B. Papandrea D. Tuz K. Anderson T. M. Jayakumar S., 2015 Spatiotemporal differences in the c-fos pathway between C57BL/6J and DBA/2J mice following flurothyl-induced seizures: a dissociation of hippocampal Fos from seizure activity. Epilepsy Res. 109: 183–196. 25524858 10.1016/j.eplepsyres.2014.11.009 PMC4272448

[bib25] Kadiyala S. B. Yannix J. Q. Nalwalk J. W. Papandrea D. Beyer B. S., 2016 Eight flurothyl-induced generalized seizures lead to the rapid evolution of spontaneous seizures in mice: a model of epileptogenesis with seizure remission. J. Neurosci. 36: 7485–7496. 27413158 10.1523/JNEUROSCI.3232-14.2016 PMC4945668

[bib61] Kadiyala, S. B., and R. J. Ferland, 2017 Dissociation of spontaneous seizures and brainstem seizure thresholds in mice exposed to eight flurothyl-induced generalized seizures. Epilepsia Open 2: 48–58. 10.1002/epi4.12031 PMC5560332 28825051

[bib26] Kandratavicius L. Alves Balista P. Lopes-Aguiar C. Ruggiero R. N. Umeoka E. H., 2014 Animal models of epilepsy: use and limitations. Neuropsychiatr. Dis. Treat. 10: 1693–1705. 25228809 10.2147/NDT.S50371 PMC4164293

[bib27] Kolisek M. Montezano A. C. Sponder G. Anagnostopoulou A. Vormann J., 2015 PARK7/DJ-1 dysregulation by oxidative stress leads to magnesium deficiency: implications in degenerative and chronic diseases. Clin. Sci. 129: 1143–1150. 26453619 10.1042/CS20150355

[bib28] Kosobud A. E. Crabbe J. C., 1990 Genetic correlations among inbred strain sensitivities to convulsions induced by 9 convulsant drugs. Brain Res. 526: 8–16. 2078820 10.1016/0006-8993(90)90243-5

[bib29] Lenzen K. P. Heils A. Lorenz S. Hempelmann A. Höfels S., 2005 Supportive evidence for an allelic association of the human KCNJ10 potassium channel gene with idiopathic generalized epilepsy. Epilepsy Res. 63: 113–118. 15725393 10.1016/j.eplepsyres.2005.01.002

[bib30] Lippert C. Listgarten J. Liu Y. Kadie C. M. Davidson R. I., 2011 FaST linear mixed models for genome-wide association studies. Nat. Methods 8: 833–837. 21892150 10.1038/nmeth.1681

[bib31] Losi G. Cammarota M. Carmignoto G., 2012 The role of astroglia in the epileptic brain. Front. Pharmacol. 3: 132. 22807916 10.3389/fphar.2012.00132 PMC3395023

[bib32] Miller, L. A., J. Gunstad, M. B. Spitznagel, J. Mccaffery, J. Mcgeary *et al.*, 2011 CAMTA1 T polymorphism is associated with neuropsychological test performance in older adults with cardiovascular disease. Psychogeriatrics 11: 135–140. 10.1111/j.1479-8301.2011.00357.x 21951953

[bib33] Morgan A. P. Fu C.-P. Kao C.-Y. Welsh C. E. Didion J. P., 2015 The mouse universal genotyping array: from substrains to subspecies. G3 6: 263–279. 26684931 10.1534/g3.115.022087 PMC4751547

[bib34] Mortazavi A. Williams B. A. McCue K. Schaeffer L. Wold B., 2008 Mapping and quantifying mammalian transcriptomes by RNA-Seq. Nat. Methods 5: 621–628. 18516045 10.1038/nmeth.1226 PMC13303166

[bib35] Mozhui K. Ciobanu D. C. Schikorski T. Wang X. Lu L., 2008 Dissection of a QTL hotspot on mouse distal chromosome 1 that modulates neurobehavioral phenotypes and gene expression (J. Flint, Ed.). PLoS Genet. 4: e1000260. 19008955 10.1371/journal.pgen.1000260 PMC2577893

[bib36] Mozhui K. Lu L. Armstrong W. E. Williams R. W., 2012 Sex-specific modulation of gene expression networks in murine hypothalamus. Front. Neurosci. 6: 63. 22593731 10.3389/fnins.2012.00063 PMC3350311

[bib37] Murray K. D. Isackson P. J. Eskin T. A. King M. A. Montesinos S. P., 2000 Altered mRNA expression for brain-derived neurotrophic factor and type II calcium/calmodulin-dependent protein kinase in the hippocampus of patients with intractable temporal lobe epilepsy. J. Comp. Neurol. 418: 411–422. 10713570 10.1002/(sici)1096-9861(20000320)418:4<411::aid-cne4>3.0.co;2-f

[bib38] Neumann P. E. Collins R. L., 1991 Genetic dissection of susceptibility to audiogenic seizures in inbred mice. Proc. Natl. Acad. Sci. USA 88: 5408–5412. 2052619 10.1073/pnas.88.12.5408 PMC51882

[bib39] Overall R. Kempermann G. Peirce J. Lu L. Goldowitz D., 2009 Genetics of the hippocampal transcriptome in mouse: a systematic survey and online neurogenomics resource. Front. Neurosci. 3: 3. 20582282 10.3389/neuro.15.003.2009 PMC2858614

[bib40] Pal S. Sun D. Limbrick D. Rafiq A. DeLorenzo R. J., 2001 Epileptogenesis induces long-term alterations in intracellular calcium release and sequestration mechanisms in the hippocampal neuronal culture model of epilepsy. Cell Calcium 30: 285–296. 11587552 10.1054/ceca.2001.0236

[bib41] Pandey K. Dhoke R. R. Rathore Y. S. Nath S. K. Verma N., 2014 Low pH overrides the need of calcium ions for the shape-function relationship of calmodulin: resolving prevailing debates. J. Phys. Chem. B 118: 5059–5074. 24779925 10.1021/jp501641r

[bib42] Papandrea D. Anderson T. M. Herron B. J. Ferland R. J., 2009 a Dissociation of seizure traits in inbred strains of mice using the flurothyl kindling model of epileptogenesis. Exp. Neurol. 215: 60–68. 18950623 10.1016/j.expneurol.2008.09.016 PMC2656999

[bib60] Papandrea, D., W. S. Kukol, T. M. Anderson, B. J. Herron, and R. J. Ferland, 2009b Analysis of flurothyl-induced myoclonus in inbred strains of mice. Epil. Res. 87: 130–136. 10.1016/j.eplepsyres.2009.08.003 PMC2788057 19744831

[bib43] Persike D. Lima M. Amorim R. Cavalheiro E. Yacubian E., 2012 Hippocampal proteomic profile in temporal lobe epilepsy. J. Epilepsy Clin. Neurophysiol. 18: 53–56.

[bib44] Samoriski G. M. Applegate C. D., 1997 Repeated generalized seizures induce time-dependent changes in the behavioral seizure response independent of continued seizure induction. J. Neurosci. 17: 5581–5590. 9204939 10.1523/JNEUROSCI.17-14-05581.1997 PMC6793817

[bib45] Schroder W. Hinterkeuser S. Seifert G. Schramm J. Jabs R., 2000 Functional and molecular properties of human astrocytes in acute hippocampal slices obtained from patients with temporal lobe epilepsy. Epilepsia 41: S181–S184. 10999541 10.1111/j.1528-1157.2000.tb01578.x

[bib46] Shinawi M. Coorg R. Shimony J. S. Grange D. K. Al-Kateb H., 2015 Intragenic CAMTA1 deletions are associated with a spectrum of neurobehavioral phenotypes. Clin. Genet. 87: 478–482. 24738973 10.1111/cge.12407

[bib47] Steinhäuser C. Seifert G. Bedner P., 2012 Astrocyte dysfunction in temporal lobe epilepsy: K+ channels and gap junction coupling. Glia 60: 1192–1202. 22328245 10.1002/glia.22313

[bib48] Tse K. Puttachary S. Beamer E. Sills G., and T. Thippeswamy, 2014 Advantages of repeated low dose against single high dose of kainate in C57BL/6J mouse model of status epilepticus: behavioral and electroencephalographic studies. PLoS One 9: e96622. 24802808 10.1371/journal.pone.0096622 PMC4011859

[bib49] Turrigiano G. G. Nelson S. B., 2004 Homeostatic plasticity in the developing nervous system. Nat. Rev. Neurosci. 5: 97–107. 14735113 10.1038/nrn1327

[bib50] Wang X. Mozhui K. Li Z. Mulligan M. K. Ingels J. F., 2012 A promoter polymorphism in the Per3 gene is associated with alcohol and stress response. Transl. Psychiatry 2: e73. 22832735 10.1038/tp.2011.71 PMC3309544

[bib51] Williams R. W. Mulligan M. K., 2012 Genetic and molecular network analysis of behavior. Int. Rev. Neurobiol. 104: 135–157. 23195314 10.1016/B978-0-12-398323-7.00006-9 PMC4267574

[bib52] Yang H. Wang J. R. Didion J. P. Buus R. J. Bell T. A., 2011 Subspecific origin and haplotype diversity in the laboratory mouse. Nat. Genet. 43: 648–655. 21623374 10.1038/ng.847 PMC3125408

[bib53] Zerr P. Martin B. Adelman J. P., 2000 The murine Bis1 seizure gene and the Kcnab2 gene encoding the beta2-subunit of the K+ channel are different. Neurogenetics 2: 231–234. 10983719 10.1007/pl00022974

